# Physiological properties of astroglial cell lines derived from mice with high (SAMP8) and low (SAMR1, ICR) levels of endogenous retrovirus

**DOI:** 10.1186/1742-4690-5-104

**Published:** 2008-11-25

**Authors:** Boe-Hyun Kim, Harry C Meeker, Hae-Young Shin, Jae-Il Kim, Byung-Hoon Jeong, Eun-Kyoung Choi, Richard I Carp, Yong-Sun Kim

**Affiliations:** 1 Ilsong Institute of Life Science, Hallym University, 1605-4 Gwanyang-dong Dongan-gu, Anyang, Gyeonggi-do 431-060, South Korea; 2 New York State Institute for Basic Research in Developmental Disabilities, Staten Island, NY 10314, USA

## Abstract

Previous studies have reported that various inbred SAM mouse strains differ markedly with regard to a variety of parameters, such as capacity for learning and memory, life spans and brain histopathology. A potential cause of differences seen in these strains may be based on the fact that some strains have a high concentration of infectious murine leukemia virus (MuLV) in the brain, whereas other strains have little or no virus. To elucidate the effect of a higher titer of endogenous retrovirus in astroglial cells of the brain, we established astroglial cell lines from SAMR1 and SAMP8 mice, which are, respectively, resistant and prone to deficit in learning and memory and shortened life span. MuLV-negative astroglial cell lines established from ICR mice served as controls. Comparison of these cell lines showed differences in: 1) levels of the capsid antigen CAgag in both cell lysates and culture media, 2) expression of genomic retroelements, 3) the number of virus particles, 4) titer of infectious virus, 5) morphology, 6) replication rate of cells in culture and final cell concentrations, 7) expression pattern of proinflammatory cytokine genes. The results show that the expression of MuLV is much higher in SAMP8 than SAMR1 astrocyte cultures and that there are physiological differences in astroglia from the 2 strains. These results raise the possibility that the distinct physiological differences between SAMP8 and SAMR1 are a function of activation of endogenous retrovirus.

## Introduction

The group of SAM strains was derived from an inadvertent cross between AKR mice and an unknown mouse strain. Although the background of the original progeny was the same, subsequent inbreeding from these progeny led to a series of senescence-prone (SAMP) and senescence-resistant strains (SAMR). Findings in the SAMP strains manifested various phenotypes which are generally different from SAMR strains [1]. Compared to the SAMR strains, SAMP strains have shorter life spans, perioptic lesions, ruffled coat and lordokyphosis. In addition to these general signs, each of the SAMP strains shows specific abnormalities [2-4]. For example, the SAMP8 strain used in the current study shows early deficits in learning and memory [5-7].

Many mouse strains have ancient genomic inserts, termed proviruses, some of which have the capacity to produce intact virions (MuLV)[8]. One of the progenitors of the SAM strains, the AKR mouse strain, expresses high levels of the prototype ecotropic endogenous retrovirus, murine leukemia virus (MuLV), which is termed Akv; the AKR strain exhibits life-long viremia with this virus [9,10]. Previous studies reported that the titer of MuLV in SAMP8 mice was higher than in SAMR1 mice, a difference that was particularly pronounced in the brain [10,11]. The capsid antigen of MuLV was seen in a number of cell types in brain, and there was extensive activation of astroglial cells [11]. The astrocytosis was seen in areas in which neurons contained MuLV antigen, and there was extensive vacuolation. Glial cells, which were once considered merely supportive elements and were thought to be passive cells in the nervous system, have recently come to central stage in efforts to understand the workings of the brain. Astroglial cells, one of the glia cell types in the central nervous system, are highly numerous and likely to have many divergent roles [12]. Morphologically astroglial cells are in closely associated with neurons and have extensive contacts with endothelial cells from capillaries [13,14]. Therefore, astroglial cells are positioned to serve as signaling pathways between neurons, between astroglial cells and between neurons and capillaries. It is also known that astroglial cells are prone to persistent infection or viral transformation [15].

To analyze the contribution of astroglial cells in the difference in MuLV titers in brains of SAMP8 and SAMR1 mice, we have established astroglial cell lines from SAMR1, SAMP8, and ICR mice to investigate functional capacity to produce MuLV particles and to provide *in vitro *cell models for studying endogenous retroviruses and their effects.

## Methods

### Animals

SAMR1 and SAMP8 mice have been maintained as inbred strains in the Institute for Basic Research animal colony and the Ilsong Institute of Life Science animal colony. Pathogen-free SAMR1, SAMP8, and ICR (Daehan Biolink, Korea) animals have been housed in cages in a clean facility. All animals are on a 12-h light, dark cycle.

### Cell culture

Zpl 2-1 and C6 cell lines were used for the neuronal cell marker and the glial cell marker, respectively. The neuronal cell line Zpl 2-1 was established from hippocampus of Zürich I mice, as previously described [16]. The glial cell line, C6, was cloned from a rat glial tumor (ATCC CCL-107). Both cell lines were maintained in DMEM supplemented with 10% FBS, 100 unit/ml penicillin and 100 μg/ml streptomycin (Gibco BRL), incubated at 37°C in 5% CO_2_.

### Establishment of astroglial cell lines from SAMR1, SAMP8 and ICR mice

Primary astrocyte cells were cultured from 1 day neonates from SAMR1, SAMP8, and ICR mice [17]. Cells were obtained from neonates in full compliance with the ethical guidelines of the National Institutes of Health (NIH). Cells were cultured on 5 μg/ml poly-L-lysine (P-L-L; Sigma)-coated dishes with culture media (DMEM with 10% FBS, 100 unit/ml penicillin and 100 μg/ml streptomycin, Gibco BRL), incubated at 37°C in 5% CO_2 _and transfected with SV40 large T antigen containing vector (φSV40; provided by Dr T. Onodera, Tokyo University) using 8 μg/ml of hexadimethrine bromide (Sigma-Aldrich, San Diego, CA, USA)[18]. After 24 h, cells were detached from culture dishes to eliminate microglia cells and oligodendrocytes and then transferred to new culture dishes. The origin of the mouse lines and characteristics of cell lines used in the present study are shown in Table 1.

**Table 1:** Mouse origin and characteristics of cell lines.

Cell lines	Mouse origin	Cell line expression
		
		MuLV mRNA and CAgag
R1A1, R1A2, R1A5	SAMR1	+
P8A1, P8A7, P8A9	SAMP8	+++
ICR-A1, ICR-A2, ICR-A3	ICR	-

### Western blot analysis and immunocytochemistry

For Western blot analysis, 50 μg protein from brain homogenates from each cell lysate and 40 μl of cell-free cell culture medium obtained after centrifugation at 25000 × g, 4°C for 30 min were separated on 12% Tris-glycine gels and transferred to nitrocellulose membrane (Amersham) [19]. The membrane was blocked with 5% nonfat dry milk in 0.1% TBST (Tris-buffered saline with tween-20; 20 mM Tris-HCl, 140 mM NaCl, 0.1% Tween-20) for 1 h at room temperature and then probed with one of the following primary antibodies: rat-anti-GFAP (glial fibrillary acidic protein) at dilution of 1:5000 (DAKO, Glostrup, Denmark), mouse-anti-NeuN (neuron-specific nuclear protein) at 1: 1000 (Chemicon, Temecula, California, USA), mouse-anti-CD11b (Integrin α M) at 1:1000 (Serotec, Oxford, UK), mouse-anti-CNPase (2',3'-cyclic nucleotide 3'-phosphodiesterase) at 1:1000 (Sigma-Aldrich, St. Louis, Missouri, USA), and goat-anti-MuLV CAgag at 1:5000 (Quality Biotech, Inc.)[11,16]. The primary antibody was incubated overnight at 4°C, and the appropriate secondary antibodies conjugated with horseradish peroxidase (Zymed, San Francisco, California, USA), anti-rat-HRP-conjugated at 1:3000, anti-mouse-HRP-conjugated at 1:5000, anti-goat-HRP-conjugated at 1:3000, were then added. Bound antibodies were visualized by chemiluminescence (Pierce, Rockford, Illinois, USA). Mouse-anti-β-actin at 1:10000 (Sigma-Aldrich, St. Louis, Missouri, USA) was used as a cellular marker. Expression levels of each protein were quantified by densitometer (GS-800, Bio-Rad, California, USA).

For immunocytochemistry, cells were plated on glass-cover slips and cultured for 24 h. Cells were fixed with 4% paraformaldehyde in PBS, permeabilized with 0.2% Triton X-100 (Sigma-Aldrich) at room temperature for 10 min, treated with 5% normal donkey serum (Jackson, West Grove, Pennsylvania, USA) in PBS at room temperature for 1 h, and then rinsed with PBS. Cells were incubated with primary antibodies against rabbit-anti-GFAP at 1:100 (DAKO) and rat-anti-GFAP at 1:100 as an astrocyte marker, mouse-anti-MAP2 (microtubule-associated protein 2) at 1:50 (Upstate, Charlottesville, Virginia, USA) as a neuronal marker, mouse-anti-CD11b at 1:50 (Serotec) as a microglia marker and mouse-anti-CNPase at 1:50 (Sigma-Aldrich) as an oligodendrocyte marker, then maintained overnight at 4°C. Appropriate secondary antibodies conjugated with fluorochromes (Zymed), anti-rabbit-FITC at 1:200, anti-rat-FITC at 1:200, anti-goat-TRITC at 1:200 and anti-mouse-TRITC at 1:200, were then applied. After washing with PBS, cells were incubated with 10 μM DAPI (4',6-Diamidino-2-phenyindole, dilactate)(Sigma-Aldrich) at 37°C for 1 min and observed using confocal microscopy (Zeiss). DAPI staining was used as a cellular marker. For double-staining, cells on cover slips were prepared as noted above and then incubated with each primary antibody at the dilution listed above overnight at 4°C. After incubation, slides were washed with PBS and then appropriate secondary antibodies conjugated with fluorochromes (Zymed) were applied at the appropriate dilution as noted above. After washing with PBS, 10 μM DAPI staining was applied and the cells observed using confocal microscopy (Zeiss). The results were representative of at least three separate experiments.

### Reverse transcriptase polymerase chain reaction (RT-PCR)

Total mRNA was extracted using Trizol reagent (Invitrogen, Carlsbad, California, USA) and cDNA was synthesized from 2 μg of total RNA by reverse transcription using AMV reverse transcriptase (Promega, Madison WI) and oligo (dT) primer. To test for integration of SV40 large T antigen, genomic DNA was extracted from cultured cells using a DNA extraction kit (Qiagen, Hilden, Germany). PCR was performed with the following primers (Bioneer, Daejon, Korea): SV40 large T antigen, sense: 5'-TGAGGCTACTGCTGACTCT-3'; antisense: 5'-GCATGACTCAAAAAACTTAGCAATTCTG-3'; Akv, sense: 5'-ATGGAGAGTACAACGCT CTCA-3'; antisense: 5'-GAGGTTAGATTGTTGCTTACTG-3'. As a cellular marker GAPDH (glyceraldehydes 3-phosphate dehydrogenase) was performed with the following primers, sense: 5'-TGGTATCGTGGAAGGACTCATGAC-3'; antisense: 5'-ATGCCAGTGAGCTTCCC GTTCAGC-3'. Expression levels of Akv and GAPDH were quantified by densitometer (GS-800, Bio-Rad, California, USA). Purified RNA (2 μg) was used as a substrate for single-stranded cDNA synthesis. An aliquot (5 μl) of the cDNA of each sample was used for PCR with primers for IFNγ, TNF-α, TNF-β, IL-1α, IL-1β, IL-6, and β-actin, a housekeeping gene. The PCR primers used are shown in Table 2[20-22]. The DNA mixture was amplified for 30 cycles (each consisting of denaturation 45 sec at 95°C, annealing for 45 sec at 58°C, extension for 1 min at 72°C), received a final extension for 10 min at 72°C and was stored at 4°C in a thermal cycler (Amplified Biosystem, USA) [21,22]. Products were analyzed by 1% agarose gel electrophoresis and visualized by ethidium bromide staining under UV light.

**Table 2:** Primer sequences for RT-PCR analysis of cytokines.

	Polarity	Sequence	Product size
IFN-γ	sense	5-CATGAAAATCCTGCAGAGCC-3	304 bp
	antisense	5-GGACAATCTCTTCCCCACCC-3	
TNF-α	sense	5-GGCAGGTCTACTTTGGAGTCATTGC-3	307 bp
	antisense	5-ACATTCGAGGCTCCAGTGAATTC-3	
TNF-β	sense	5-TGGCTGGGAACAGGGGAAGGTTGAC-3	205 bp
	antisense	5-GTGCTTTCTTCTAGAACCCCTTGG-3	
IL-1α	sense	5-CTCTAGAGCACCATGCTACAGAC-3	308 bp
	antisense	5-TGGAATCCAGGGGAAACACTG-3	
IL-1β	sense	5-TTGACGGACCCCAAAAGATG-3	203 bp
	antisense	5-AGAAGGTGCTCATGTAATCA-3	
IL-6	sense	5-GCCAGAGTCCTTCAGAGAGAT-3	213 bp
	antisense	5-CCGAGTAGATCTCAAAGTGAC-3	
iNOS	sense	5-GTCGACCTTCCGAAGTTTCTGGCAGCAGCG-3	470 bp
	antisense	5-GTCGACGAGCCTCGTGGCTTTGGGCTCCTC-3	
β-actin	sense	5-TGTGATGGACTCCGGTGACGG-3	198 bp
	antisense	5-ACAGCTTCTCTTTGATGTCACGC-3	

IFN, interferon; TNF, tumor necrosis factor; IL, interleukin; iNOS, inducible nitric oxide synthase

### Culture of cell lines for UV plaque assay

SC-1 cells (ATCC CRL-1404) were grown in Dulbecco's modified eagle medium (DMEM) + 10% fetal bovine serum (FBS) + 100 unit/mL penicillin + 100 μg/mL streptomycin (DMEM10A). The XC cell line (ATCC CCL-165) was grown in DMEM + 10% FBS without antibiotics (DMEM10). SC-1 and XC cells were harvested for use in plaque assays using trypsin and suspended in the appropriate medium for assay. All cell growth and plaque assays were done at 37°C in a 5% CO_2 _incubator.

### Preparation of cell homogenates for UV plaque assay

Cells were harvested by trypsinization and kept on ice until further processing by homogenization in DMEM (10% w/v), using 20 strokes in a hand-operated tissue homogenizer. Serial dilutions of cell homogenates were prepared in DMEM + 5% FBS + penicillin-streptomycin + 25 μg/mL DEAE-dextran (DMEM5A-DEAE).

### SC-1 UV plaque assay

Ecotropic MuLV was quantitated using the SC-1/UV plaque assay [10]. SC-1 cells were plated onto 60 mm dishes at 10^5 ^cells/dish in 4 mL DMEM10A. The next day, 1 h before the addition of cell homogenates, medium in the dishes was discarded and replaced with 3 mL DMEM5A-DEAE. One mL of sample (brain homogenate or cell line homogenate) diluted in the same medium was then added to plates. One to 2 days after addition of homogenates, medium was removed and replaced with 4 mL/dish DMEM5A. After 5 days, medium was removed and cultures were exposed to 30 s of UV irradiation. Immediately after UV irradiation, 4 mL of a suspension containing 3.0 × 10^5 ^XC cells/mL in DMEM10 were pipetted into each dish. After 24 h incubation, the medium was discarded. Cultures were washed once with phosphate-buffered saline (PBS), fixed with 100% methanol for 5 min and stained with hematoxylin (Fisher Scientific, USA) for 5 min. Hematoxylin was discarded, the cultures washed twice with tap water, and the plaques counted under a dissecting microscope.

### Electron microscopy

Cells were fixed in half-Karnovsky's fixative (1.66% glutaraldehyde, 1.6% paraformaldehyde buffered with 0.1 M cacodylate buffer) for 2 h at 4°C. Post-fixation was done in 1% osmium tetroxide buffered with cacodylate buffer for 1.5 h at 4°C. Following fixation, cells were pelleted and washed three times in 0.1 M cacodylate buffer. Cell pellets were dehydrated through a graded ethanol-propylene oxide series and embedded in Epon 812 (Electronic Microscopy Science). Ultra thin sections (75 nm) were cut in a RMC MTXL ultramicrotome (Tucson, USA) and stained with 2% uranyl acetate and lead citrate. The sections were observed using a transmission electron microscope (Zeiss-EM109, Oberkochen, Germany).

For immuno-electron microscopy experiments, cells were fixed for 3 h at 4°C with 3% paraformaldehyde and 0.25% glutaraldehyde in phosphate buffer (0.1 M, pH 7.4). Pellets were dehydrated in increasing concentrations of ethanol and embedded in LR white (Electron Microscopy Sciences, Hatfield, PA, USA) and cured for 48 h at 50°C. Ultra thin sections (75 nm) were collected on nickel grids and used for labeling with a goat antiserum specific for the virus CAgag protein, followed by incubation with a rabbit anti-goat IgG conjugated to 15 nm gold particles (Electron Microscopy Sciences, Hatfield, PA, USA). Sections were post-stained with 2% uranyl acetate and lead citrate and observed with a transmission electron microscope (Zeiss-EM109, Oberkochen, Germany). The viral particles were counted and their diameters measured in 13 independent fields (85 μm^2^) of R1A and P8A cell lines. The number of gold particles were counted in 18 independent fields (85 μm^2^) within the plasma membrane.

To obtain negative staining images, cells were scraped then frozen and thawed for three cycles. Supernatant and disrupted cells were combined and centrifuged at 1000 × g for 10 min at 4°C. Supernatants were transferred to a 20% sucrose cushioned tube and centrifuged at 130000 × g for 3 h at 4°C (SW28 rotor, Beckman). Viral particles were suspended in 30 μl of sterile phosphate-buffered saline (PBS) then incubated at 37°C for 30 min. Fixation and staining were done using 2% phosphotungustic acid (PTA) solution.

### Estimation of the cell growth cycle and morphological analysis

Each of the cell lines was seeded at a density of 1 × 10^5 ^cells and incubated at 37°C in 5% CO_2_; a series of separate cell cultures were stained with 0.4% trypan blue solution (Sigma-Aldrich, USA) and counted each day for 12 days using hemacytometer (Sigma-Aldrich, USA)(Kim et al., 2005). Cell growth and morphological analysis was assessed using inverted microscopy (Zeiss, Oberkochen, Germany). Each cell count and microscopic analysis was repeated at least three times.

### Statistical analysis

Statistical analyses were performed by appropriate one-way ANOVA test. All data were reported as means ± SD. A *P *value of less than 0.05 was considered significant.

## Results

### Analysis of expression of the MuLV gene and protein in the brains of ICR, SAMR1 and SAMP8 mice

**Figure 1:**
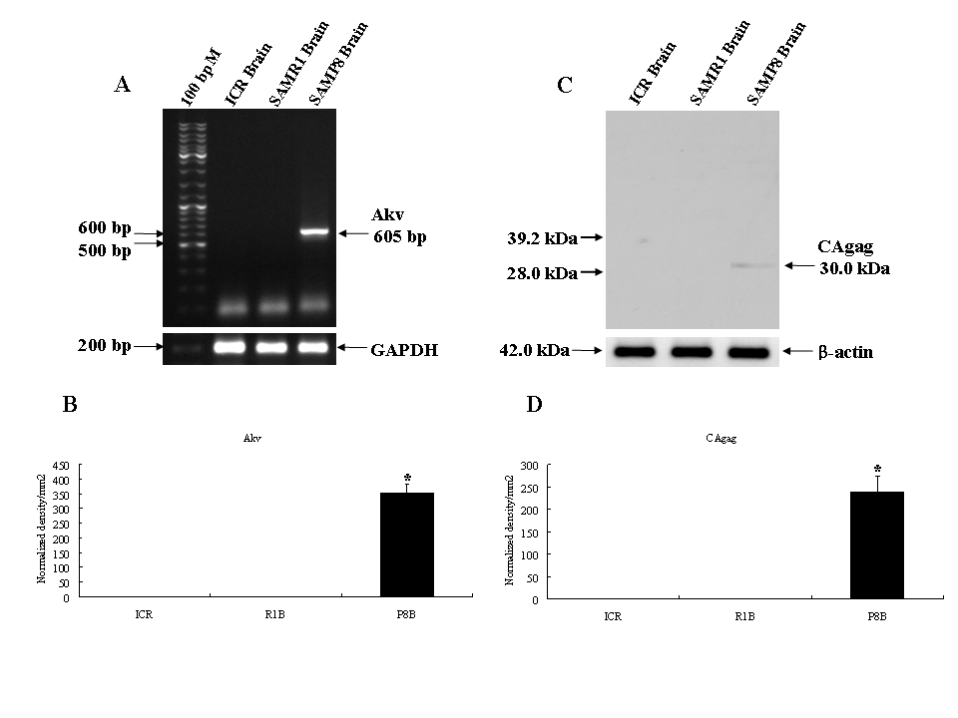
**Expression levels of the MuLV gene and protein in ICR (n = 12), SAMR1 (n = 12) and SAMP8 (n = 10) brains**. (A) Analysis of genetic expression level of the MuLV, Akv gene (605 bp), by RT-PCR in the brains of ICR, SAMR1, and SAMP8 mice. Levels of GAPDH served as a measure of sample concentration. (B) Densitometry analysis of Akv expression in A. *statistically significant difference (*p *< 0.01). (C) Analysis of protein expression levels of the MuLV, CAgag (30 kDa), by Western blot in the brains of ICR, SAMR1, and SAMP8 mice. 50 μg of brain homogenates were used. Levels of B-actin were used as a measure of sample concentration. (D) Densitometry analysis of CAgag expression in C. *statistically significant difference (*p *< 0.01).

The expression levels of the MuLV gene and protein were investigated in brains of 12 months ICR, SAMR1, and SAMP8 mice. Using RT-PCR analysis of endogenous MuLV gene expression, we have shown that the expression of MuLV was not detected in ICR nor SAMR1 brains (R1B) but was present in SAMP8 brains (P8B) (Fig. 1A). MuLV gene expression level in P8B was significantly higher than ICR and R1B (*p *< 0.01) (Fig. 1B). To analyze the expression level of the MuLV protein, CAgag, a Western blot was performed. In agreement with RT-PCR data, MuLV protein was neither detected in ICR nor R1B but was present in P8B (*p *< 0.01) (Fig. 1C and 1D). GAPDH and β-actin were included as concentration monitors for RT-PCR analysis and Western blot, respectively.

### Astroglial cell lines were established from SAMR1, SAMP8 and ICR mice

**Figure 2:**
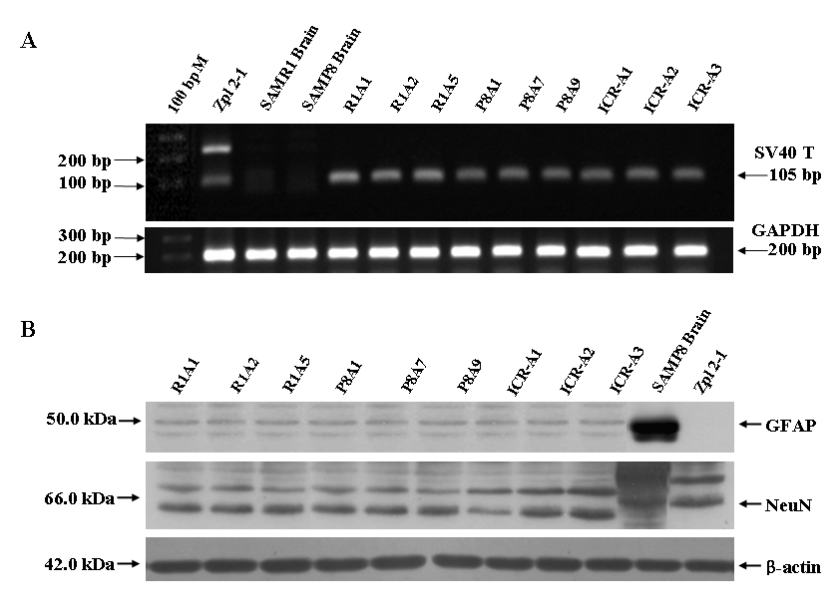
**Establishment of astroglial cell lines immortalized by SV40 T antigen**. (A) Confirmation of SV40 large T antigen (105 bp) in R1A, P8A, and ICR-A cell lines by PCR analysis. 100 bp M: 100 bp DNA ladder marker; Zpl 2-1: a positive control; SAMR1 and SAMP8 brain: negative controls. (B) Characterization of cell types by Western blot analysis. SAMP8 brain: a positive control for GFAP (50.0 kDa) and NeuN (66.0 kDa); Zpl 2-1: a positive control for NeuN and a negative control for GFAP.

**Figure 3:**
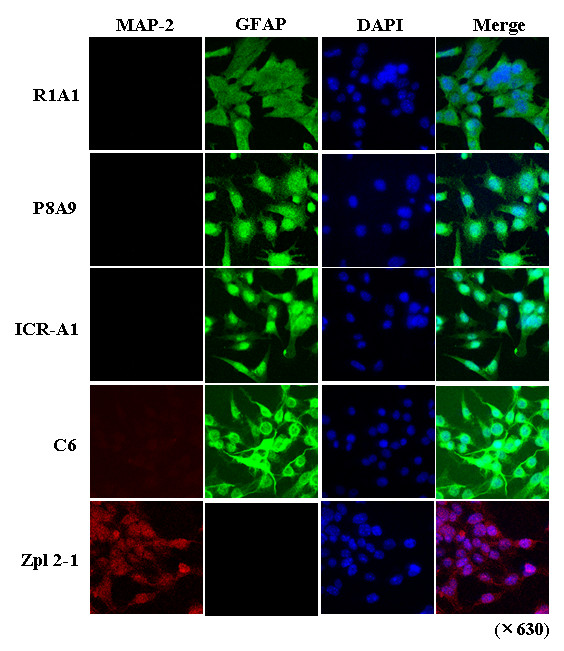
**Confirmation of cell-type of established astroglial cell lines by immunocytochemistry**. Characterization of cell types by immunofluorescence analysis. DAPI staining (blue fluorescence) was used as a cellular marker. Astroglial cell marker GFAP: green fluorescence; neuronal cell marker MAP-2: red fluorescence. C6 and Zpl 2-1 cell lines were used for positive glial cell control and positive neuronal cell control, respectively.

Three distinct astrocyte cell lines were established from the cerebral region of SAMR1 (R1A cell lines: R1A1, R1A2 and R1A5), SAMP8 (P8A cell lines: P8A1, P8A7 and P8A9), and ICR (ICR-A cell lines: ICR-A1, ICR-A2 and ICR-A3) mice (Table 1). The presence of the gene for SV40 large T antigen was examined with PCR analysis using GAPDH (glyceraldehyde-3-phosphate dehydrogenase) as the housekeeping control gene. As shown in Fig. 2A, established R1A, P8A, and ICR-A cell lines had similar expression levels of SV40 large T antigen as an immortalization marker. The cell-type marker antibodies described in Materials and Methods were used to characterize the transformed cell lines. SAMP8 mouse brain was positive for both the astroglial (GFAP) and the neuronal (NeuN) marker. Zpl 2-1 and C6 cell lines were used as positive controls for the neuronal marker and the astroglial marker, respectively. R1A, P8A, and ICR-A cell lines were astroglial-positive in Western blot analysis using antibodies against GFAP, a 50 kDa protein band which did not react with Zpl 2-1 hippocampal neuronal cell lysates (Fig. 2B). Using anti-NeuN antibody, a 66 kDa protein band was detected only in Zpl 2-1 cell lysates and SAMP8 brain homogenates (Fig. 2B). Thus, the established cell lines were shown to be composed of astroglial cells. The results of immunofluorescence analysis were in accordance with the Western blot findings: R1A, P8A, ICR-A, and C6 cell lines were positive for GFAP staining but not MAP-2 staining, whereas Zpl 2-1 cells were positive for MAP-2 but not GFAP (Fig. 3). All cultures were negative for the oligodendrocyte and microglial markers in both Western blot and immunofluorescence experiments (data not shown).

### Expression of the MuLV gene and protein in R1A, P8A and ICR-A cell lines

**Figure 4:**
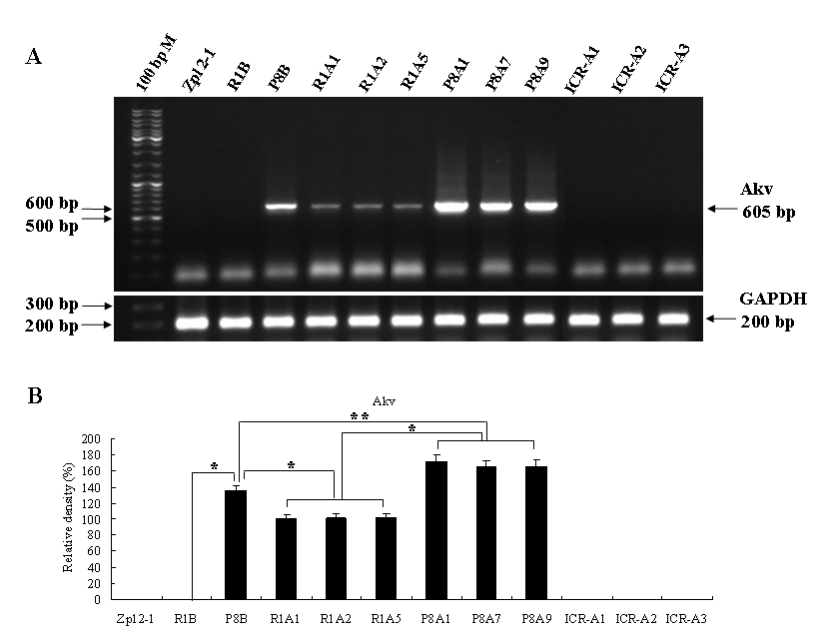
**Genetic expression level of the MuLV in R1A, P8A and ICR-A cell lines**. (A) mRNA expression of Akv (605 bp) in R1A, P8A, and ICR-A cell lines. As a housekeeping gene GAPDH (200 bp) was used. 100 bp M: 100 bp DNA ladder marker; Zpl 2-1 and R1B (12-month-old SAMR1 brain): negative controls; P8B (12-month-old SAMP8 brain): a positive control. (B) Densitometry of Akv gene expression levels in the ICR-A, R1A and P8A cell lines. Expressed levels of MuLV in P8A cell lines were significantly higher than in R1A cell lines. *statistically significant difference (*p *< 0.01).

RT-PCR analysis was used to assess the level of endogenous MuLV expression in R1A, P8A and ICR-A cell lines. Homogenate of SAMP8 brain was used as a positive control and Zpl 2-1 cell line and SAMR1 brain homogenate served as negative controls. We found that MuLV expression was not detected in ICR-A or Zpl 2-1 cell lines, nor in SAMR1 brain (R1B) but was present in R1A and P8A cell lines and SAMP8 brain (P8B) tissue (Fig. 4A). The expression level of MuLV in P8A cell lines was significantly higher than in R1A cell lines (*p *< 0.01) (Fig. 4B). In contrast, GAPDH was expressed to the same extent in all samples.

**Figure 5:**
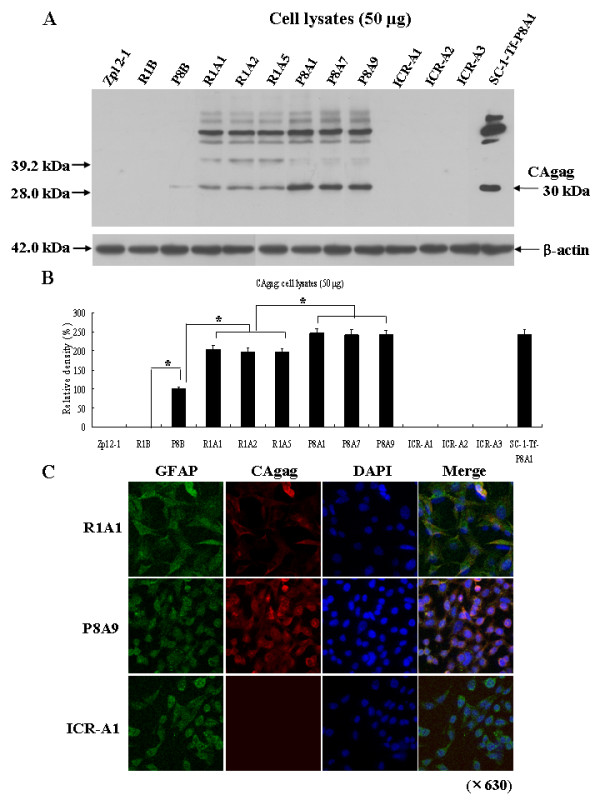
**Expression levels of the MuLV protein, CAgag, in ICR-A, R1A and P8A cell lines**. (A) Western blot analysis for expression of CAgag in cell lysates (50 μg) using anti-CAgag antibody. β-actin was used as a sample concentration marker. Zpl 2-1 and R1B (12-month-old SAMR1 brain): negative controls; P8B (12-month-old SAMP8 brain) and SC-1-Tf-P8A1 (SC-1 cells infected with P8A1 cell homogenate): positive controls. (B) Densitometry of CAgag and β-actin in the ICR-A, R1A and P8A cell lines. CAgag protein levels were significantly higher in the P8A cell lines than in the R1A cell lines. *statistically significant difference (*p *< 0.01). (C) Immunofluorescence analysis of CAgag in ICR-A, R1A and P8A cell lines. Expression of CAgag in R1A, P8A and ICR-A cell lines was analyzed using anti-GFAP (green) and anti-CAgag (red) antibodies. DAPI staining (blue fluorescence) was used as a cellular marker.

In order to analyze the expression of the MuLV protein, CAgag, Western blot analysis was performed. CAgag was detected in SAMP8 brains, R1A cell lines, P8A cell lines, and in SC-1 cells which had been infected with P8A cell line homogenate (labeled SC-1-Tf-P8A1), whereas CAgag was not detected in the Zpl 2-1 cell lines, SAMR1 brains, and ICR-A cell lines (Fig. 5A). The CAgag levels in P8B cell lines were significantly higher (*p *< 0.01) than in R1A cell lines (Fig. 5B). SAMR1 brain homogenate and Zpl 2-1 cell homogenate served as negative controls and SAMP8 brain homogenate was used as a positive control. The same amount of total protein was analyzed as shown by the similar levels of β-actin expression in all of the samples. In agreement with the Western blot findings, the R1A and P8A cell lines were positive for CAgag immunostaining, whereas ICR-A cell lines were negative (Fig. 5C). Staining for endogenous MuLV protein was detected in cytoplasm where it was merged with GFAP staining. In accordance with Western blot analysis, the amount of CAgag staining was less in R1A cell lines than in P8A cell lines. The difference in color of the merge pictures seen for the R1A1 and P8A9 is probably a function of the fact that the concentration of CAgag is higher in the P8 cell lines than in the R1 cell lines.

### Release of CAgag protein and MuLV particles from R1A and P8A cell lines and analysis of infectivity levels

**Figure 6:**
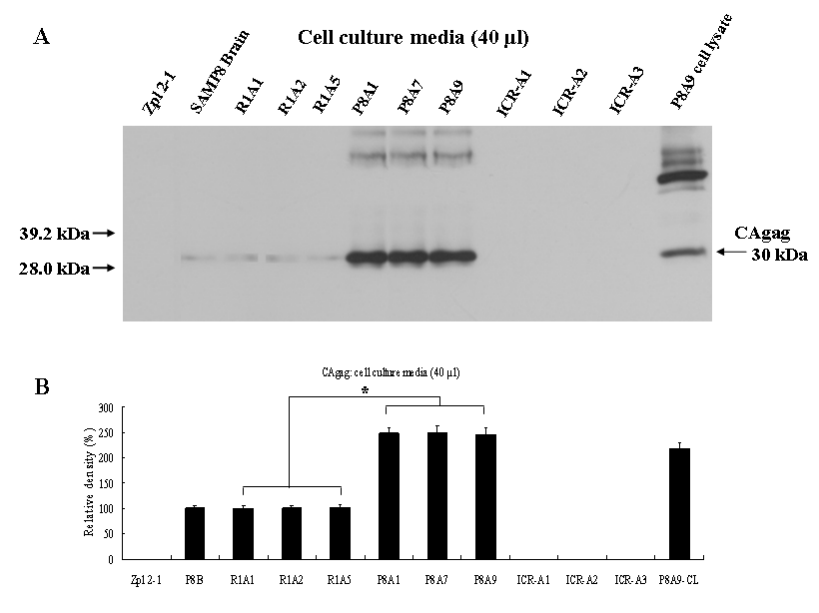
**Immunoblot analysis of CAgag in culture media from ICR-A, R1A and P8A cell lines**. (A) Western blot analysis for expression of CAgag (40 μl of culture media) using anti-CAgag antibody. Zpl 2-1 culture media: a negative control; SAMP8 brain (12-month-old) and P8A9 cell lysate (P8A9-CL) (50 μg): positive controls. (B) Densitometry of CAgag in cell culture media. CAgag protein levels in P8A cell culture media were significantly higher than in R1A cell culture media. *statistically significant difference (*p *< 0.01).

To determine whether cell-associated CAgag detected in homogenates of R1A and P8A cells was released from cells, culture media of each cell line was harvested and analyzed using anti-CAgag antibody in Western blots. CAgag was detected in R1A cell lines at low levels and in P8A cell lines at high levels (Fig. 6A). There was a significant difference in the level of released CAgag between the two types of cell lines (*p *< 0.01) (Fig. 6B). The level of CAgag found in P8A9 cell lysate (P8A9-Cl in Fig. 6B) was slightly less than that seen in the media from P8A9 cell culture. Zpl 2-1 culture media was used as a negative control and SAMP8 brain homogenate was the positive control.

**Figure 7:**
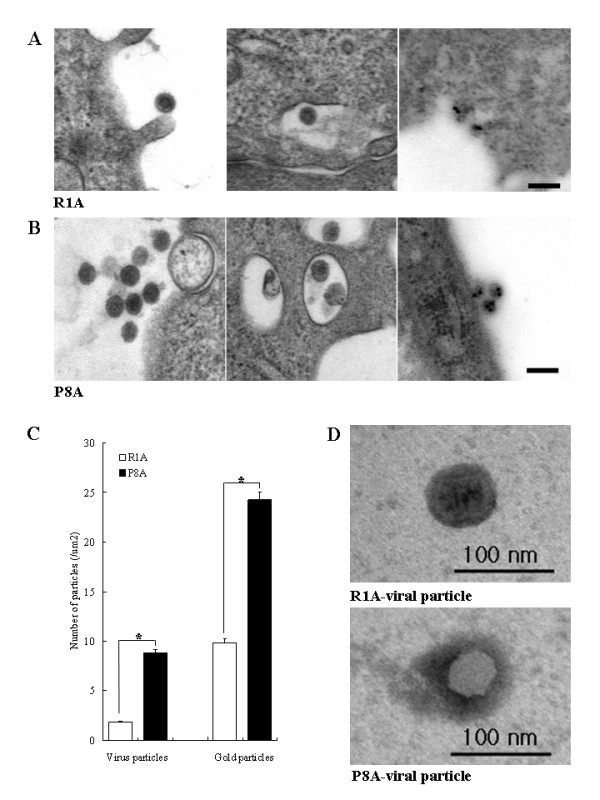
**Electron microscopy of viral-like particles generated by the R1A and P8A cell lines**. (A) Images of virus particles from R1A cell lines. Single virus-like particles were present at the cell membrane (left panel) and at an intracellular vacuole (middle panel). Immuno-gold image was obtained after labeling with an antibody specific for the virus CAgag protein (right panel) (Scale bar = 400 nm). (B) Images of the virus particles from P8A cell lines. Multiple virus-like particles were present at the cell membrane (left panel) and in intracellular vacuoles (middle panel). Immuno-gold image was obtained after labeling with anti-CAgag antibody (right panel) (Scale bar = 400 nm). (C) Quantification of virus particles and of labeling of viral particles with gold beads. The average number of viral particles was 1.85/μm^2 ^and 8.85/μm^2 ^in the R1A and P8A cell lines, respectively. The average number of gold particles was 9.98/μm^2 ^and 24.28/μm^2 ^in the R1A and P8A cell lines, respectively. *statistically significant difference (*p *< 0.01). (D) Negative stain **i**mages of single viral particle from R1A and P8A cell lines. R1A-viral particle size averaged 70 nm and P8A-viral particle averaged 80 nm large.

Using transmission electron microscopy (TEM), synthesized virus particles budding from the plasma membrane were observed in R1A and P8A cell lines (Fig. 7A and 7B); similar particles were not observed in ICR-A cell lines (data not shown). The concentration of MuLV particles was significantly higher in P8A cell lines than in R1A cell lines (*p *< 0.01), as determined by either uranyl acetate/lead citrate staining or by gold-labeling (Fig. 7C). The diameters of particles in the fixed tissues were measured in 13 independent fields (85 μm^2^), and each experiment was repeated three times. Viral particles observed in R1A cell lines averaged 70 nm including their envelope, whereas particles from P8A cell lines averaged 80 nm.

For PTA-stained virus preparations derived from the different cultures, the different morphology seen in virus particles from R1A and P8A shown in Fig. 7D is not a consistent difference between the two cell cultures, but rather reflects the variation seen in PTA preparations: the R1A particle morphology shown in Fig. 7D can also be seen in P8A preparations and vice-versa.

Testing of R1A, P8A, and ICR-A cell lines for ecotropic MuLV infectivity showed striking differences in virus titer among these cell lines (Table 3). R1A and ICR-A cell lines exhibited no demonstrable virus in either cells or supernatants even using undiluted inoculum, whereas 10^4^~10^6 ^plaque-forming units (PFU)/ml of virus was found in P8A cells and supernatants.

**Table 3:** Ecotropic MuLV in astrocyte cell lines and supernatant of SAMR1, SAMP8 and ICR mice.

Cell lines	Infectivity*	
	
	Cells	Supernatant
R1A1	-	-
R1A2	-	-
R1A5	-	-
P8A1	+	+
P8A7	+	+
P8A9	+	+
ICR-A1	-	-
ICR-A2	-	-
ICR-A3	-	-

* Plaque-forming units (PFU)/ml

+ indicates a titer higher than 10^4^/ml.

- indicates no demonstrable virus even in undiluted material.

### Different characteristics of astroglial cells in the R1, P8 and ICR cell lines

**Figure 8:**
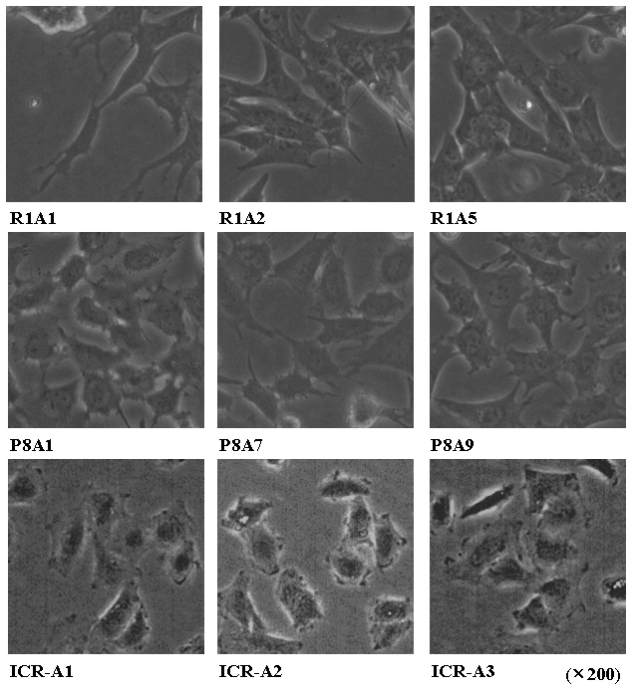
**Characterization of morphology of established cell lines**. Morphological appearance of the R1A, P8A, and ICR-A cell lines were compared using an inverted microscope. P8A cells showed ruffled edges of plasma membranes in contrast to the comparatively smooth edges seen in R1A and ICR-A cells.

The cell morphology of the 3 types of cells is different: The R1A cells have a long hexagonal shape with smooth plasma membranes, P8A cells have a pentagonal shape with ruffled plasma membranes, and ICR-A cells have a pentagonal shape with smooth plasma membranes (Fig. 8).

**Figure 9:**
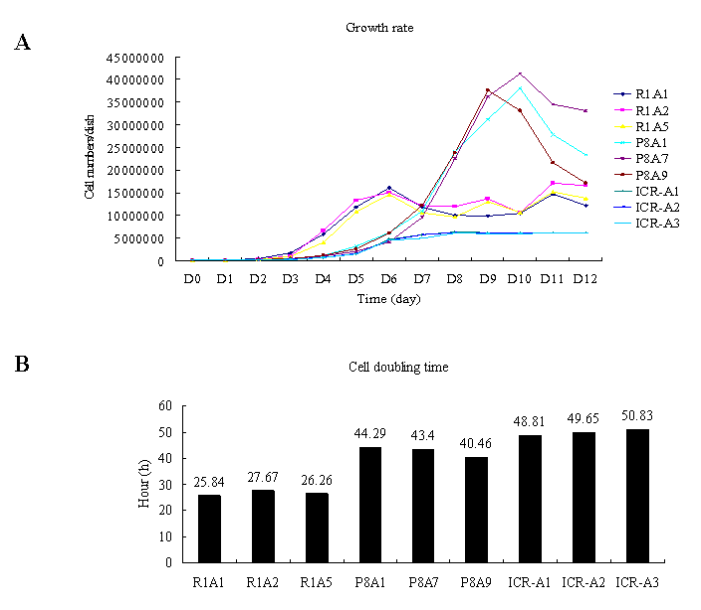
**The difference in growth rates of R1A, P8A and ICR-A cell lines**. (A) Comparison of the growth rates of R1A, P8A, and ICR-A cell lines. (B) Representative doubling times of each cell line. Proliferation time was estimated from the growth rate of each cell line. The numbers above each column represent the doubling time in hours of each cell line.

R1A, P8A, and ICR-A cell lines also showed differences in their growth rates. For the first 7 days, the proliferation rates for the R1A cell lines were higher than those for the P8A and ICR-A cell lines (Fig. 9A); this was reflected in an analysis of the doubling times for the cell lines (Fig. 9B). The growth rates for P8A and ICR-A cell lines were virtually identical prior to D7 and, therefore, their doubling rates were similar. After D7, cell numbers for R1A and ICR-A cell lines remained relatively constant, whereas the P8A cell lines showed a dramatic increase in cell counts until D10 and then decreased on Days 11 and 12 (Fig. 9A).

### Expression profiles of genes coding for proinflammatory cytokines in R1A, P8A and ICR-A cell lines

**Figure 10:**
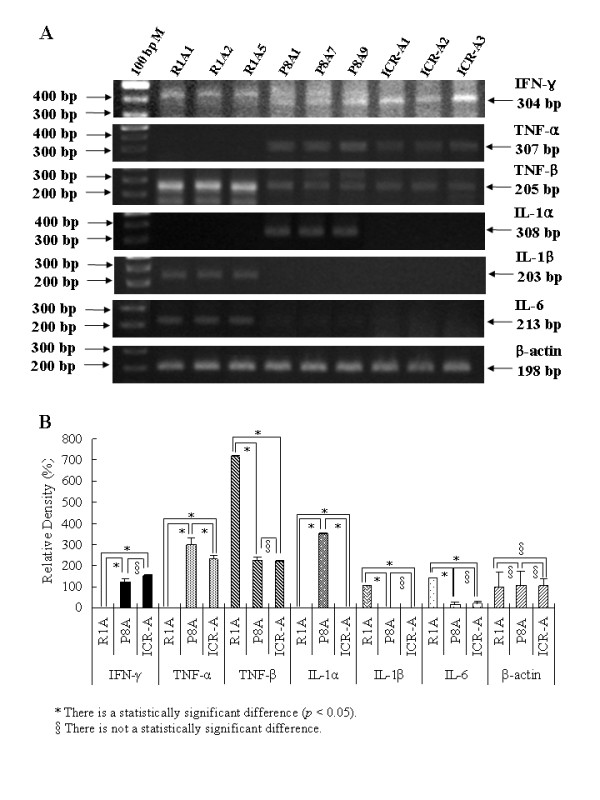
**RT-PCR analysis of inducible proinflammatory cytokine genes expressed in R1A, P8A and ICR-A cell lines**. (A) Proinflammatory cytokine genes, TNF-α, TNF-β, IL-1α, IL-1β and IL-6 plus the anti-viral cytokine gene, IFN-γ, were assessed in each cell line. β-actin was used for analysis of cell protein concentration. 100 bp M, 100 bp DNA ladder marker. (B) Quantitative analysis of proinflammatory cytokine genes and the gene for IFN-γ. Expression levels were measured by densitometry. Expression of the housekeeping gene, β-actin, was measured in each preparation. *statistically significant difference (*p *< 0.05). §Comparisons not showing a statistically significant difference.

The levels of gene expression of the following inducible cytokines were measured in the 3 cell types: IFN-γ, TNF-α/β, IL-1α/β, IL-6 and iNOS. The results of RT-PCR analysis showed marked induction of TNF-β, IL-β, and IL-6 in R1A cell lines, IFN-γ, TNF-α, and IL-1α in P8A cell lines, and IFN-γ, TNF-α and TNF-β in ICR-A cell lines (Fig. 10A). Expression levels were quantitated and compared by densitometry (Fig. 10B); iNOS was not activated in any astroglial cell lines used in this study (data not shown).

Significant differences in expression between R1A and P8A cell lines were seen in IFN-γ, TNF-α and TNF-β, IL-1α, IL-1β, and IL-6 (Fig. 10B). Significant differences between P8A and ICR lines were seen for TNF-α and IL-1α.

## Discussion

The extensive studies of endogenous retroviruses have centered primarily on their dramatic effects as exemplified by the indication of leukemia-lymphoma in several mouse strains, e.g. AKR [23]. The examination of cryptic effects induced by endogenous retroviruses in other species and in mice with different genetic characteristics has received less attention. Recently, there have been several studies aimed at understanding the expression of endogenous retroviruses and their pathological and physiological effects [23,24]. In humans, it has been reported that the human endogenous retrovirus (HERV-W) is highly expressed in the central nervous system (CNS) glia of individuals with multiple sclerosis (MS) [23,25,26]. Endogenous retroviruses have been described in other mammals and in birds; it was suggested that some types of cancers are induced by endogenous retroviruses in these species [27]. The SAMP8 mouse strain expresses MuLV and develops several pathophysiological changes including neurodegeneration that is characterized by astrocytosis and neuronal loss [2,23,28].

The SAMR1 mouse that is characterized by low or no MuLV infectivity, normal histological appearance, normal life span and normal capacity for learning and memory served as the contrasting strain for SAMP8. The ICR strain was the virus negative control. Analysis of the transformed astroglial cell lines derived from SAMR1, SAMP8 and ICR mice revealed a number of differences: (1) The initial (through day 6) growth of the 3 R1A cell lines exceeded the rate for both the P8A and ICR-A lines, however, the P8A cell lines replicated faster in the subsequent 6 days and reached higher concentrations than the other cell lines, (2) Although all of the cell lines were shown to be composed of astroglia, there were morphological differences; of particular interest in this regard is the ruffled plasma membranes of the P8A lines vs. the smooth membranes of the R1A and ICR-A lines, (3) The expression of a number of cytokines were significantly different in P8A vs. R1A lines: IFN-γ, TNF-α and TNF-β, IL-1α, IL-1β, and IL-6. For a number of these cytokines, the level of expression of RNA was not a function of MuLV titer, since although there was a significant difference between P8A lines and R1A lines, there was no difference between P8A and the virus-free ICR-A lines; this was the finding for IFN-γ, TNF-β, IL-1β and IL-6.

We postulate that one potential cause of different morphological appearance and proliferation rates in the cell lines derived from the 3 mouse strains is their different expression levels of MuLV. The viral expression level could affect control mechanisms in cells and their metabolic activity. Subsequently, the different metabolism may affect morphological appearance and cell proliferation rates [29]. It is, of course, possible that other factors during the development of the cell lines affect their morphology and physiological characteristics.

The expression of MuLV with regard to the virus messenger RNA and CAgag protein was highest in the P8A cell lines and absent from ICR-A cell lines. Expression in R1A cell lines was significantly less than in P8A lines. These data correlate with the findings in SAMR1 and SAMP8 brains in which the latter reveal high expression compared with the low level or absent expression seen in R1 brains.

Electron microscopy showed particles budding from plasma membranes of both P8A and R1A. These particles were similar in appearance although there was a small difference in size. There were, however, significantly higher numbers seen in P8A cultures than in R1A cultures; these findings correlated with the levels of released CAgag in the P8A vs. R1A cell lines. Despite the observation of virus particles in R1A cultures, there was no infectivity present (nor was there in ICR-A cultures), however, high levels of MuLV infectivity were found in P8A cultures. In order to determine why there was no infectivity in R1A cultures despite the presence of virus particles, an assessment of the protein sequences of the envelope proteins of virus produced in R1A and P8A cultures would be of interest.

A comparison of expression levels between cell cultures and brain homogenates reveals a number of unexpected findings. It is clear that expression levels in the transformed SAMP8 cell cultures are higher than those found in SAMP8 brain. As examples, CAgag in cell lysates (Fig. 5) are higher than in SAMP8 brain; also, the expression of MuLV RNA in SAMP8 cell lines is significantly greater than in SAMP8 brain (Fig. 4). There are a number of possible explanations for this result: the cell cultures are composed of a single cell type; these cells have been transformed and probably have an altered level of expression of various macromolecules compared to normal brain cells in vivo. Furthermore, cultured cells are harvested at a specific time after subculturing so that the cells proceed in their cell cycles at a comparable rate. In contrast, the brain is composed of a variety of cell types which are not transformed and are at various stages of replication or are quiescent. Also, although all assayed samples contain similar levels of housekeeping RNA (Fig. 4) and protein (Fig. 5), the brain contains macromolecules such as myelin, which would be inert with regard to retrovirus replication. The above differences reduce the value of comparisons between expressions in brain homogenate vs. cell culture lysates.

SAMR1 mice do not contain the Emv11 provirus that codes for the Akv1 virus present in AKR and SAMP8 mice. In the current study, no expression of Akv1-specific RNA was seen in brains of SAMR1 mice, however, there was low level of expression in the R1A cell lines. The explanation for this finding is not clear. We do know that there are non-Emv11 proviruses in SAMR1 mice and some of these yield low levels of infectious MuLV [2,10]. The transformation process might augment the expression of these SAMR1 endogenous retroviruses which may share primer sequences with the primers used in our PCR experiments.

A major consideration in this study was the role of MuLV-expressing astroglial cells in the killing of neurons. Several neurodegenerative changes in Alzheimer's disease (AD), Parkinson's disease (PD), HIV-associated dementia (HAD), and prion diseases are explained by generation of neurotoxins, mainly inducible nitric oxide (iNOS) from glial cells [30,31]. Astroglial cells in healthy brain do not express iNOS however iNOS is induced in both mice and humans following viral infections [31]. Many proinflammatory cytokines associated with the innate immune system induce iNOS expression in astroglial and microglial cells. IL-1β and IFN-γ can each induce iNOS in glial cells. Other cytokines such as TNF-α/β usually induce iNOS in conjunction with IL-1β or IFN-γ [32,33]. The failure to observe iNOS induction in SAMP8 mice is surprising but is probably related to the variation seen in cytokines that induce iNOS in different cell types and culture conditions [31]. The fact that we did not observe expression of iNOS in P8 indicates that induction of neurodegeneration in this strain is accomplished via an alternate pathway. It is possible that the physiological changes seen in P8A cell lines are a function of the level of expression of either TNF-α and/or IL-1α in that these are the cytokines in P8A that differ significantly from levels found in both R1A and ICR-A cell lines.

The fact that P8A astrocyte cultures express high levels of MuLV antigen and infectivity is significant in that there is strong astrocytic activation in the proximity of CAgag-positive neurons in SAMP8 mice [11]. The caveat concerning this point is that the cell cultures are transformed, whereas the in situ astrocytes are normal. However, if astrocytes in brains of SAMP8 mice can express MuLV antigen and infectivity, questions arise concerning the role of these astrocytes in the pathology and clinical changes seen in SAMP8 mice. The use of the SAMP-SAMR model system has recently been applied to analysis of the pathogenesis and treatment of neurodegenerative diseases [34,35] and could prove useful in analysis of the relationship between virus presence and various genetic factors that lead to diseases such as Alzheimer's disease [36].

In future studies, we will examine neuronal cell cultures derived from SAMR1, SAMP8 and ICR strains. The results obtained with these neuronal cell lines will be compared to the findings with the astrocyte cell lines from these mouse strains.

## Competing interests

The authors declare that they have no competing interests.

## Authors' contributions

BHK performed the RNA manipulation, RT-PCR, cell culture, immunocytochemistry, and Western blot analysis. HCM performed the viral plaque assay. HYS performed the electronic microscopy. RIC provided the animals. BHJ directed RT-PCR. EKC, RIC and YSK directed the whole experiments and manuscript. All authors read and approved the final manuscript.
